# Patients' perspectives on self-testing of oral anticoagulation therapy: Content analysis of patients' internet blogs

**DOI:** 10.1186/1472-6963-11-25

**Published:** 2011-02-03

**Authors:** Syed Ghulam Sarwar Shah, Ian Robinson

**Affiliations:** 1 Multidisciplinary Assessment of Technology Centre for Healthcare (MATCH), Department of Information Systems and Computing, Brunel University, Uxbridge, Middlesex, UB8 3PH, UK; 2 Centre for the Study of Health and Illness, School of Social Sciences, Brunel University, Uxbridge, Middlesex, UB8 3PH, UK

## Abstract

**Background:**

Patients on oral anticoagulant therapy (OAT) require regular testing of the prothrombin time (PT) and the international normalised ratio (INR) to monitor their blood coagulation level to avoid complications of either over or under coagulation. PT/INR can be tested by a healthcare professional or by the patient. The latter mode of the testing is known as patient self-testing or home testing. The objective of this study was to elicit patients' perspectives and experiences regarding PT/INR self-testing using portable coagulometer devices.

**Methods:**

Internet blog text mining was used to collect 246 blog postings by 108 patients, mainly from the USA and the UK. The content of these qualitative data were analysed using XSight and NVivo software packages.

**Results:**

The key themes in relation to self-testing of OAT identified were as follows: Patient benefits reported were time saved, personal control, choice, travel reduction, cheaper testing, and peace of mind. Equipment issues included high costs, reliability, quality, and learning how to use the device. PT/INR issues focused on the frequency of testing, INR fluctuations and individual target (therapeutic) INR level. Other themes noted were INR testing at laboratories, the interactions with healthcare professionals in managing and testing OAT and insurance companies' involvement in acquiring the self-testing equipment. Social issues included the pain and stress of taking and testing for OAT.

**Conclusions:**

Patients' blogs on PT/INR testing provide insightful information that can help in understanding the nature of the experiences and perspectives of patients on self-testing of OAT. The themes identified in this paper highlight the substantial complexities involved in self-testing programmes in the healthcare system. Thus, the issues elicited in this study are very valuable for all stakeholders involved in developing effective self-testing strategies in healthcare that are gaining considerable current momentum particularly for patients with chronic illness.

## Background

The development of the effective empowerment of patients through "more rights and control over (their) health and care" has been strongly suggested in recent years [1], which could provide increased control and choice to patients regarding the selection of a particular therapy, and an appropriate healthcare setting as well as the use of a particular medical device technology. By these means, patients could take control of and manage some key health related issues themselves. These might include important aspects of self-caring, self-monitoring and self-management. For example, self-monitoring of oral anticoagulation therapy by patients themselves could lead to their greater independence with less inconvenience [2].

### Anti-coagulant therapy

Oral anti-coagulant therapy (OAT) is indicated for several medical conditions such as atrial fibrillation (AFib), mechanical heart valve prostheses, deep vein thrombosis (DVT), pulmonary embolism (PE), and stroke [3]. It has however been particularly recommended for patients and especially the elderly who may have or be at risk of having a stroke or AFib, to prevent and treat thromboses [4]. The OAT has a narrow optimum range designed to prevent the major adverse health effects of both over- and under- coagulation; therefore, regular monitoring of OAT, both in terms of the dose and the frequency of administration, is required [5]. The monitoring of OAT is done by checking the prothrombin time (PT) and the international normalised ratio (INR) of the patient taking an oral anticoagulant. These two terms: PT and INR are important from the clinicians' and patients' perspectives because they show the blood coagulation level; therefore, they provide a measure of whether the blood coagulation level is stable (which is generally called the therapeutic or target level and thus is desirable) or variable (whether it has gone up or down with the extent of variation determining the need for medical intervention). Thus, it is important that the context of both PT and the INR terms be set out.

### Prothrombin Time

The prothrombin time is the time taken, in seconds, for the patient's plasma to clot in an extrinsic pathway of coagulation, which is most commonly measured for monitoring OAT through warfarin for example [6]. The reference values for PT may vary between laboratories [7].

### International Normalised Ratio

The World Health Organisation's expert committee on biological standardization [8] has defined the international normalised ratio through the following calculation:

INR = (PT patient/MNPT)^ISI^

Where PT patient is the PT for the patient's blood; MNPT is the mean normal prothrombin time (MNPT) of the healthy adult population, and ISI is the international sensitivity index (ISI) of the system [3].

An INR value of 1 is regarded as normal. Though a precise target INR value for each medical condition may be somewhat different, an overall target INR value of 2.5 is recommended for most medical conditions requiring OAT [5].

### OAT monitoring

The PT and the INR of patients stable on OAT is usually checked at the healthcare facility by healthcare professionals or at home by the patient or by his/her care provider. Monitoring in hospitals is undertaken, usually in an anticoagulation clinic or in an haematology department, at 4-6 week intervals [5] while the frequency of monitoring of PT/INR at home may vary from one patient to another.

In the case of self-testing, the patient is informed by his/her general practitioner or the anticoagulation clinic doctor or nurse about the target therapeutic range of his/her (patient's) PT/INR. Consequently, the patient tests his/her PT/INR level and reports the results to his/her clinician, who interprets the PT and the INR values, adjusts the dose of the oral anticoagulant drugs, and informs the patient about the new dosage. Self-testing of the INR and the PT is performed with a hand held device known as coagulometer.

### Coagulometers

Coagulometer devices are broadly divided into two categories i.e. monitors for patient self-testing, which are small, portable and require capillary whole blood taken by finger pricking, and monitors for professional use, which are large and multifunctional [4]. Generally the former type of monitors are intended for the patient's use (either by patients themselves or by their carers) while the latter type of monitors are intended for use by healthcare professionals such as haematology doctors and nurses in a primary, secondary or tertiary healthcare facility. At present, there are a number of commercially available coagulometers and some of them have been specifically evaluated in the UK [5] and a few types of coagulometer devices have been approved for home use in the USA [3].

Recently, there has been increase in the availability and use of coagulometer devices for testing the PT and the INR of patients taking oral anticoagulants such as warfarin [9]. These devices can be used in different settings (e.g. hospitals in both primary and secondary care), care homes, private homes and at any place) and by different types of users (e.g. healthcare professionals, patients and carers) [10]. However, the effectiveness of this type of device varies and depends on the user (operator), environment and circumstances in which they are used [11]. The settings in which the device is used and characteristics of users, particularly those of patients, may have important implications for the safe, effective and intended use of the device. This is because OAT is given in several medical conditions such as AFib, mechanical heart valve prostheses, DVT, PE, and stroke [3] The needs and requirements of such patients might be very different due to differences in not only in their medical conditions but differences in their age, gender, culture, skills, training, physical abilities, cognition and capabilities [12,13]. The study of the needs and requirements of different types of end users of coagulometer devices for home use would thus help in understanding various important issues with respect to self-testing of OAT.

The overall aim of this study was to elicit patients' perspectives and experiences on self-testing of PT/INR using portable coagulometer devices.

## Methods

### Blogs as a data source

The Internet, particularly in relation to blogs, has emerged as an important source of information on various issues relating to health, medical therapy and medical device technologies. 'Blog' is a shortened form of weblog, which stands for wee-blog, and is defined as a webpage that is regularly updated with postings by blogger(s) in a dated order [14,15]. Blogs can provide first person narratives from different communities such as patients and their lay carers who otherwise may be infrequently assessed through some conventional methods [16].

Through blogs, patients can share their experiences and exchange information with other people regarding various aspects of health and healthcare such as the illness, medication and medical devices that can be used for self-testing and diagnostic purposes [17].

Due to the valuable information available on patients' blogs, a number of studies have used patients' blogs as a primary source of data, mainly qualitative in nature, on patients' perspectives regarding different medical conditions. For example, studies have been undertaken of patients with Alzheimer's disease [18], with pro-anorexia [19,20], with sexual health problems [21] and with various types of cancers [22-24]. There are however both advantages and limitations in using the Internet as a source of data [24]. The advantages of using the Internet include it being an easy, quick and inexpensive tool for collecting actual primary research data, through online and email surveys [25], and the ability to compile already available data existing in different forms such as text, audios, videos, images and so on [20]. However, most bloggers use nicknames or remain anonymous, and whilst it is difficult to identify them personally, one of the strengths of the data from blogs is that the anonymity of the bloggers is normally assured [26]. Other advantages of using the Internet as a data source can include the greater protection of human subjects through the process of anonymity, and through these publicly available documents, the avoidance of the cumbersome and time-consuming process of acquiring formal ethical approval for involving patients in direct primary research [16]. This however does not mean that using data from the Internet is devoid of ethical considerations.

In fact, a number of ethical considerations concerning the reporting of data obtained from the Internet have already been discussed and reported at great length by others [27-29]. There is however a growing consensus among researchers that if Internet data is freely and publicly accessible then it can be used for considered research without prior approval [21,24]. On this premise, data taken from the Internet have in fact been widely used already [24].

We however acknowledge that there are a number of limitations of using the Internet blogs as a research data source. The limitations may include the use of the nicknames and a feigned identity, age, gender and place of location by the bloggers. This might be due to the fact that bloggers want to be especially certain of their anonymity [30,31]. It is therefore very difficult to verify bloggers' demographic details [32] and thus be able to compare findings with a comparative population sample. In addition, one may argue that the issue of biased sampling may arise in using Internet blogs because access to the Internet is not available universally across all ages, gender and social classes [33]. However, the digital divide is decreasing and the access to the Internet is increasing among increasing strata of the population, such as both young and old people, as well as men and women, especially in developed countries such as the United Kingdom (UK). For example, a recent survey of access to the Internet in the UK revealed that the Internet was used in the last three months by 79% of men and 75% of women of all ages including 72% of people aged 55-64 years and 32% of people aged ≥65 years [34]. The same survey reported that the Internet was used (almost) daily by 74% of 55-64 year-olds and 59% of those aged ≥65 years [34]. In addition, the Internet was used for social networking and blogging by 44% of men and 42% of women, including 19% of 55-64 year-olds and 8% of those ≥65 years [34]. Another important issue in analysing the Internet data from patients' perspectives is the difficulty in selecting 'real' patients, and identifying and excluding bloggers who may have a commercial interest for participating in blog discussions. For example, there is evidence that commercial companies are using blogs to elicit customers' views on their products [15,35]. In such a situation, a way forward may be the careful swotting of both very negative and very positive blog posts about the theme or the product (in our case a handheld medical device for self-testing of blood coagulation level). This is the approach we adopted in identifying what we considered to be a realistic picture of patients' views on the self-testing of blood coagulation levels through the Internet blog posts. Other limitations of data from blogs can be the arrangement of blog postings in chronological order rather than on the basis of the importance and content [36]; therefore, more relevant blog postings might not be retrievable easily. Sometimes, information provided in blogs can be erroneous and misleading [37], which can raise issues of the quality of blogs data [26]. Therefore, researcher(s) need to separate blogs containing factual information from those containing misleading information, which can be verified by a survey of the relevant academic literature beforehand.

### Data collection procedure

#### Selection of blogs

The Google blogs search engine was used to find patients' blogs on self (home) PT/INR testing. The Google blog search was selected and used for a number of reasons. For example, it allowed facilities such as searching blogs and posts by title, by numbers of words, or at least one word, by the exact phrase, as well as by URL, authorship, language and date. A filtering facility for safe searching to avoid retrieving any malicious blog post or other material was also available.

#### Inclusion criteria

Blogs and blog postings written in the English language from January 2000 to October 2009 by any patient or lay carers were selected. Another criterion for the selection of blogs was that they should be currently active.

#### Exclusion criteria

Blogs and posts written and posted by people other than patients, such as manufacturers, clinics, healthcare professionals and others were excluded. Marketing articles or promotional materials regarding PT/INR testing were also excluded. No research studies based on primary and secondary research including randomised controlled trials and literature reviews were included. Blogs that were currently inactive were also not included.

#### Keywords

The following keywords and phrases were used to search the relevant blogs: INR, patient, anticoagulation, monitoring, self-testing, point of care testing, oral anticoagulant, home, warfarin therapy (Table 1). These keywords were word searched using 'AND' and 'OR' connectors. The keywords were searched first in the blog title and then in the post title, followed by 'the exact phrase', and then 'with all of the words' and 'with at least one of the words' in the blog and the post. We did not search for blogs and posts written by any particular author; therefore, this option available in the Google blog search was left blank. We did not list blogs under specific URLs; thus, this option was also not used during searches.

**Table 1:** Keywords and search hit results using Google blog search

Keywords	Hit results
***In blog title***	
INR	49
INR AND Testing	3
INR AND patient	5
INR AND patient AND selftesting	1
INR AND patient AND self testing	2
self INR testing	1
patient INR testing	1
point of care testing AND INR	3
INR AND point of care testing	1
home INR	1,663
home INR testing	13
***In blog posts***	
Home INR testing	8
"INR self testing"*	5
"INR home testing" *	2
"INR home monitoring"*	2
"anticoagulation self monitoring" *	1
"oral anticoagulant monitoring"*	1
"anticoagulation monitoring"*	91
"anticoagulation self monitoring"*	1
"warfarin therapy"*	1,398
In post: "warfarin therapy"* + In post title: patient	22
In post: "warfarin therapy"* + In post title: patient testing	5
In post: "warfarin therapy"* + In post title: home testing	11
In post: "warfarin therapy"* + In post title: self testing	1
In post: testing INR + In post title: self	16
In post: testing INR + In post title: patient	31
In post: testing INR + In post title: home	86
In post: testing INR + In post title: patient blog	7
In post: testing INR + In post title: self blog	3
In post: testing INR + In post title: blog	213
In post: "INR home testing"* + In post title: blog	2
In post: "home testing" * + In post title: blog.	3
***In posts and blog title***	
In post: "warfarin therapy"* + In blog title: patient	2

Total	3653

*Searched as a phrase

#### Search results

Searches of the aforementioned keywords resulted in 3653 hits (Table 1). After deleting duplicates and irrelevant hits, 246 relevant blog postings that were posted by 108 patients, mainly from the USA and the UK, were collected from different blogs, mainly in discussion groups on PE, DVT, heart valve replacement (HVR) and PT/INR self-testing equipment.

A blog on PT/INR self-testing equipment provided 50 postings of which 12 were posted from January 2005 to August 2005 and 38 were posted from January 2008 to October 2009. A blog on HVR through different threads linked to the blog provided 111 posts between August 2005 and October 2005. A blog on home anticoagulation monitoring provided 12 posts uploaded from January 2009 to March 2009. A blog on pulmonary embolism contained 31 posts in two different threads i.e. 'PT/INR home test' had 25 posts placed from December 2008 to January 2009 and 'fluctuating INR' had six posts uploaded from January 2008 to September 2009. Another blog on home INR monitoring provided seven posts dated from March 2008 to April 2008. Thirty-five postings dated from February 2008 to January 2009 were collected from different threads to a blog on deep vein thrombosis. In total, 246 postings were collected from the six blogs.

#### Ethical issues

Blogs that we accessed were from open access sites and did not require any formal approval to access to the content of the postings available there; however, postings required registration to take part in the discussion and submit a post. As the content of these blogs was in the open public domain, we therefore did not seek any approval prior to accessing the content of these blogs, a strategy agreed by other academic researchers [24].

We collected textual data from the open access Internet blogs without the prior consent of the bloggers to study their viewpoint and experiences regarding self-testing of OAT. However, we obtained the ethics approval from the Research Ethics Committee at the Department of Information Systems and Computing at our University. The ethics committee advised us that the range of ethical implications were far more restricted compared to most patient based research because we were analysing information that was already placed in the public domain, which meant that the information being reported in this study was secondary data. The Research Ethics Committee however observed that the data should be reported anonymously. We have therefore reported the data anonymously and at the group level rather than at an individual level.

#### Data analysis

**Figure 1:**
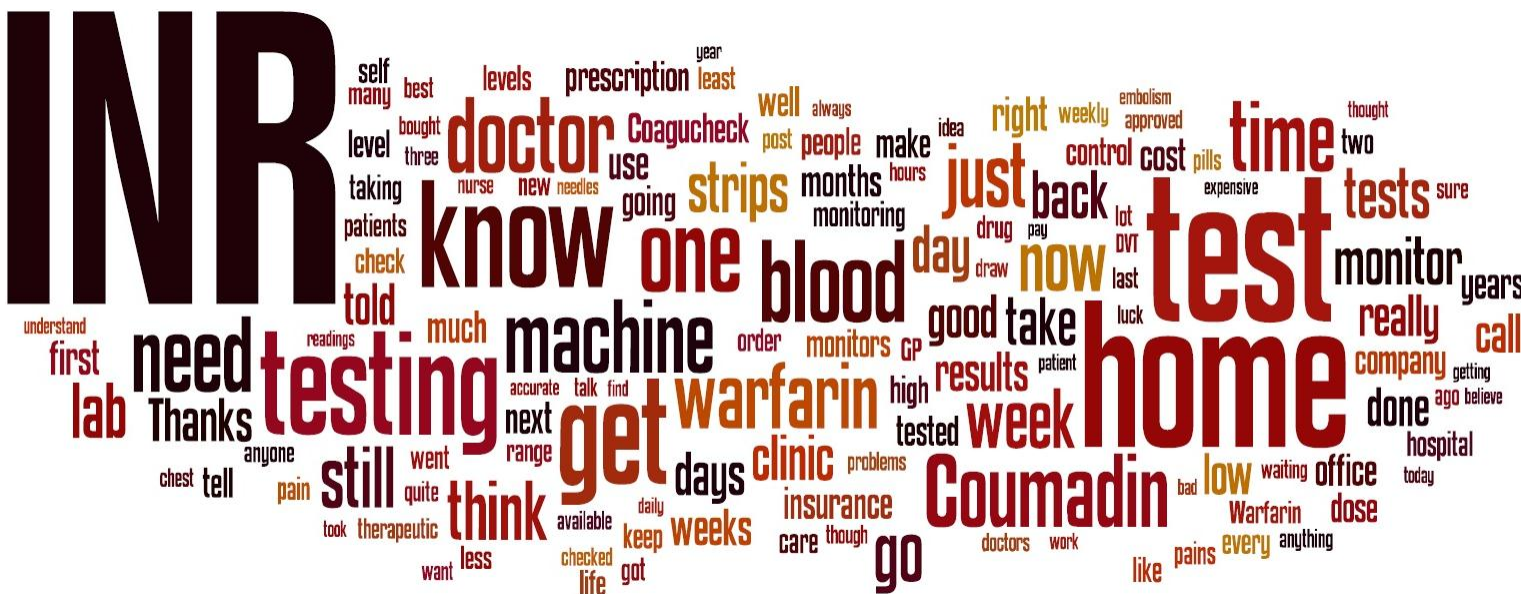
**Most commonly used words in patients' blogs on self-testing of oral anticoagulation therapy**.

**Figure 2:**
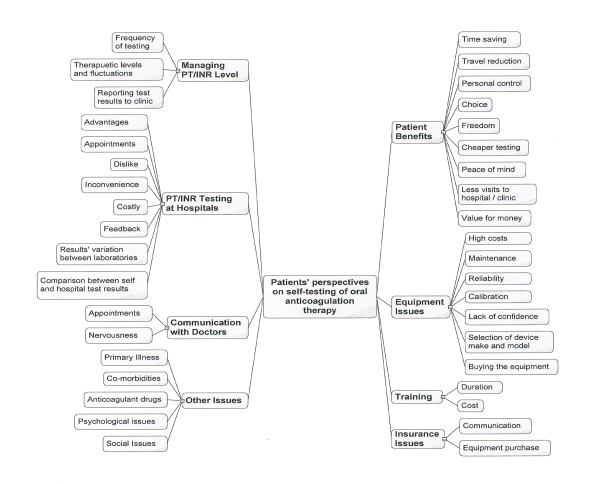
**Map of patients' perspectives on self-testing of oral anticoagulation therapy reported in patients' Internet blogs**.

Qualitative research methods are required in situations that require in depth investigation and an understanding of process to determine the exact nature of the issue being investigated, especially when the data are available in non-numeric form e.g. through text or through visual means [38]. We undertook qualitative content analysis of the data, which we collected in text form from blog postings. Validation of qualitative content analysis can be achieved through using software packages [39]; in this case we used two software packages i.e. XSight version 2 ( trial version) and NVivo version 8 (http://www.qsrinternational.com) to analyse the data because qualitative data analysis cannot easily be undertaken using single piece of software [38]. Another advantage of computer assisted content analysis of textual data is the coding reliability that helps in generating comparable results [40]. The data analysis was undertaken in a number of stages. As a first step, all data from selected blogs were saved as html files along with the details (e.g. nicknames) of bloggers. In the second step, data from the html files were saved as a single file in Microsoft word. In the third step, all data were imported into NVivo software and the frequency of most commonly used words was ascertained after removing the details of bloggers, dates, numbers and the most commonly used English words. Thereafter, a word cloud of the most commonly used words in the blogs' text was created as shown in Figure 1 (This was created in the Wordle - a Java applet, which is available at http://www.wordle.net/). This process helped us in identifying some key terms and themes relevant to blood coagulation testing as well as ensuring the suitability and relevance of the text of the blogs for the study objectives. In the fourth step, the text of all blog postings was read and issues such as the benefits, limitations, safety, training, cost, purchasing, maintenance and communications with healthcare providers were noted and a map of these issues (Figure 2) was created in XSight software, which helped in categorising the findings of this study.

## Results

The formal demographics, location and other details were not available for all bloggers from blog postings that were collected for this study; therefore, this information, helpful though it might have proved, is not reported here.

Qualitative analysis of the content of the collected blog postings resulted in the identification of seven themes, which, in no particular order, were on patient benefits, equipment related issues, managing PT/INR, laboratory testing, interaction with healthcare providers, insurance and social issues (Figure 2). Findings for each of the themes are presented as follows.

### Patient benefits

The benefits from self-testing were indicated to be time saved, travel reduction, personal control, choice and freedom, cheaper testing, and peace of mind. In reporting the benefits of having a self-testing coagulometer, one of the UK based participants wrote that the device would save his time as well as avoid a journey to the clinic for testing that he would not be able to make because of his ill health. He stated that it would also reduce the costs of the NHS (National Health Service - public sector healthcare providing service in the UK) due to fewer appointments. Reporting self-testing advantages such as cheaper testing and freedom of testing anywhere, a patient wrote that he has bought a self-testing device because he was going on holiday to a country where the cost of one PT/INR test was between £30 and £40.

A number of participants did not want to go to the hospital clinic for PT/INR testing for various reasons. For example, a female patient who did not like to travel to the clinic for testing wrote that she did not like going to see her doctor every other day therefore she would like to have a coagulometer and perform the PT/INR testing at home. Similarly, another patient, who had a HVR, wrote that if the opportunity were given then she would like to perform home testing compared to going every couple of weeks to a clinic for the testing because it was time consuming. A participant who was on heparin for a year wrote, "It would have been so useful to have a home machine, instead of having to visit the labs all the time." Another participant was interested in self-testing because she had to wait for at least two hours every week during testing at the clinic, which she did not like.

Given the freedom of self-testing with a portable coagulometer, a patient said that he wanted to buy the device because his INR could become suddenly very unstable and he could not get to the hospital because of ill health. In addition, this patient liked self-testing because it is undertaken by finger pricking and he disliked hospital based testing which requires drawing venous blood by needles, which he cannot handle.

A participant in favour of having a self-testing device wrote, "I can also check at any time, especially if feeling ill." Another wrote, "I was so excited to be doing home testing and having control of over this part at least." Given the possibility of self-testing over a longer period, a patient wrote that he had been performing self-testing for about two years. However, one of the participants was surprised to know about the possibility of self-testing. She wrote, 'I had no idea such a service (i.e. INR self-testing) was available."

One of the participants wrote that having a device is good value for money. Another patient believed that self-testing was the future. However, one of the participants wrote that she was thrilled on getting a device but became less confident after getting it.

### Equipment issues

Issues raised relating to the PT/INR self-testing device were high cost, reliability and quality, calibration, lack of confidence in accuracy, different makes and models, places to buy devices, and training in how to use the device.

The most frequently cited issue in relation to the equipment was the cost of the device and the test strips that were needed. A number of participants wrote that the device was expensive. They even compared the cost of a coagulometer with a glucometer and said that the former was more expensive then the latter. A participant wrote that the device cost plus the maintenance cost was a lot to spend for a service which can be provided free by a community nurse visiting at home. Another participant wrote that when the device price comes down she would do self-testing. This showed that some patients might be interested in PT/INR self-testing but are held back due to the high cost of the equipment. Another participant wrote that the cost incurred on the device can be claimed back effectively through paying less tax and from an insurance claim. This might be context specific, in that these points are more particular to individual healthcare systems funded in different ways, such as that in the USA compared to the UK. Regarding test strips, a participant said that he could get the strips though a clinician's prescription, while another participant wrote that he was waiting to hear from his doctor about the test strips, which he thought were very expensive.

A number of participants wrote that home testing was more expensive than the alternatives. One of them said that the cost of single home test would be about US$ 9.00 and another wrote that the cost of self-testing could be high depending on the frequency of testing.

One participant wrote that he has a second hand device, which he bought at half price. However, there was no discussion on the performance and reliability issues in using a second hand device. Nonetheless, in raising the issues of the reliability of self-testing devices, one of the participants asked whether there was any accuracy problem.

A few participants wrote that the devices need to be calibrated and one takes his device to the laboratory for checking every six months. Another wrote that he took his device to his cardiologist's office and they took several weeks to get it right.

One of the participants asked 'is there only one manufacture making them?" A few participants replied and some said there was only one manufacturer and others said there were a number of the manufacturers. A participant asked, "What is the best brand/model to use?" In response to this question, the names of a few makes/models were mentioned. One of the participants wanted to know where one could buy the best device. A few participants replied that they bought the devices directly from the manufacturers.

### Training

Regarding training in how to use an INR testing device and how to perform a test, one of the participants wrote that he had some literature and a demonstration video but he thought he still needed further training, which was going to cost him about £120, though this was less than his anticoagulation clinic would charge. Another participant wrote that training is provided; however, the details of the training provider were not mentioned. Nevertheless, a patient wrote "absolutely brilliant and easy" about the use of the device. A female participant however said that she does not have confidence in the results of home testing because the results were different from laboratory results.

### Laboratory testing

A few participants wrote about their experience of INR testing at hospital clinics and laboratories. One participant who attended an anticoagulation clinic wrote, "I ... hated it.....Also had to wait at least 2 hours to be seen every week." Another wrote that having INR test at a lab was inconvenient as well as costly. The same participant also mentioned that getting feedback from the lab about what to do next was also difficult. A participant wrote on his frustration about the cancellation of his lab test appointment, changes in testing frequency and anticoagulant dose as well as getting a new appointment and medication and contacting the doctors, the pharmacy and the laboratory.

However, some of the participants were in favour of INR testing at a lab as one participant wrote that having INR tested at the lab is advantageous because they control many more variables - which were however not described. One patient wrote that the results of INR testing by herself were about 0.4 higher compared to the lab results. The same participant also wrote that INR test results vary between laboratories. Patients compared PT/INR results of self-testing with laboratory based testing. In this regard, a participant wrote that soon after having an INR tested at the lab he performed self-testing to check the accuracy between the two.

### Managing PT/INR

Issues in managing PT/INR comprised the frequency of testing, fluctuations and the target (therapeutic) INR level and communicating INR self-test results.

#### Testing frequency

A female participant with a HVR wrote that she was testing every week at home and every three weeks at laboratory. While another patient said, "I won't be testing daily - probably 1-2/week may be spaced out further if I ever get my levels stable." A patient who had PE wrote that her PT/INR was tested twice a week while the other patient suffering from same medical condition was going to the clinic in the next four weeks for the testing. A male participant with DVT was tired of weekly testing. He wrote, "Next INR test in two week, finally! Nuff of this once a week test." Conversely, a participant wanted to test more frequently but her clinic did not want her to do that. On this situation, she wrote, "I get tested once a week, and I have had nurses/doctors tell me I can wait btwn (between) 2-4 wks (weeks) to test again, but I don't feel comfortable."

#### INR Level

Participants exchanged their views on normal and individual therapeutic levels of the INR. One of them wrote, "I think 'normal' is 1." Another wrote that the range of his INR was 2-3. A male participant with PE wrote that the therapeutic rage of his INR has to be between 2.5 and 3.5 but it had never been at the required level in the last 13 months. While another participant wrote, "I am almost always in 2-3 range."

Writing on fluctuations in the INR level, a participant wrote "I have noticed that the first (test) of spring or fall - it tends to go up to high or down to low." Participants wrote about the effect of diet especially green salads, vegetables high in vitamin K and the wine on the INR level. Given frequent fluctuations and INR levels up to 4.6, one participant wrote, "I'm wondering if it wouldn't be a good idea to have a home testing machine."

In addition, participants wrote about oral anticoagulants dosage adjustment in the light of INR fluctuations. One of the participants was unable to determine how to adjust Warfarin dosage. On this situation, a participant wrote, "They (anticoagulation clinic) adjust the dosage if INR was high or low" and another suggested that, "Dosing is based on the lab results."

#### Communicating INR results

A participant wrote that, "My clinic calls me and reminds me to take my INR test the day before it is due". He further wrote that after performing a home test, "I call them (anticoagulation clinic) back with the results the following day." Another female patient with HVR wrote that she keeps her cardiologist's office updated with home PT/INR test results. While a participant wrote that, she informs her clinic about home test results only when the readings change so that they can adjust the dose. Regarding keeping a record of self-testing results, a participant wrote that she maintains a diary as a memento.

### Interaction with healthcare providers

With regard to difficulty in communicating with doctors in managing and testing OAT, a participant suggested that patients do not deserve extreme stress and they can change the doctors if they are not communicating well. In addition, some of the participants wrote that they feel nervous when they have an appointment with their haematologist or doctor.

### Insurance issues

Patients exchanged views on the difficulty in interactions with insurance companies', especially in acquiring the self-testing equipment for themselves (patients). Some of the participants reported a long waiting time in getting a device through their insurance provider. A number of patients reported that their insurance covers purchase of a testing device and the test strip, which was however not universal but dependent on the patient's medical condition. However, some of the participants were not knowledgeable about this issue such as a patient with HVR asked, "Does your center promote the use of these (self-testing devices) for prosthetic valve patients? I was approved because of my valve."

### Other issues

Blogs participants discussed on some other issues such as their main illness and co-morbidities, anticoagulant drugs, social and psychological issues as follows.

#### Primary medical conditions and co-morbidities

Participants informed each other about the disease(s) that they had. A male participant wrote, "I have afib (Atrial fibrillation)." Another wrote, "I too am living with AFib." The same patient wrote, 'I am on Coumadin secondary to atrial fib/flutter and prosthetic valve." One of them wrote, "I had a Mechanical Mitral Valve implanted a couple of years ago." A male participant who went to a hospital due to back pain, which he described as nasty, wrote, "That's when I discovered my PE (pulmonary embolism)." A female participant wrote, "I'm glad I at least know what caused my DVT and how to manage it now/prevent another." Some of the participants had more than one disease as one of them wrote that she was hospitalised for a PE but also had liver and kidney problems.

#### Anticoagulants

Participants exchanged views on various types of anticoagulant drugs and their dosage. A patient having a PE wrote that the dose of her anticoagulation therapy had tripled in 3 months. While another participant said that, she had stopped taking an anticoagulant drug and her INR level dropped to 1.5, which according to her was below her therapeutic range of 2. Consequently, she was worried and asked other participants whether this fall in the INR was OK! One of the participants replied that the drug would not raise the INR but it would be better to talk to the doctor.

Given the possibility of being on anticoagulant therapy for life, a participant was frustrated and wrote, "I am on Warfarin (Coumadin) for life. Does it make some sense?" One of the participants replied that extra precautions were required while taking it. Another participant wrote that one of his friends had been taking this drug for the last ten years and monitoring the INR and had no problems; however, he cautioned that the drug needs close attention. One of them wrote that patients who are sensitive to the oral anticoagulants for example Warfarin, might be given other anticoagulants such as Heparin. In addition, a participant wrote that she has heard about a new drug in the market that requires less monitoring. The name of the new drug was however not reported.

#### Social and psychological issues

The participants raised a number of social and psychological issues. For example the pain, the stress, distress and disappointment that they faced during PT/INR self-testing, taking anticoagulant drugs, acquiring a coagulometer device, visiting an anticoagulation clinic/laboratory as well as communicating with their doctors, nurses, pharmacists and the insurance companies in relation to their anticoagulant therapy and its monitoring. A participant wrote that she felt her whole body was clotted after taking on oral anticoagulants. Another patient with PE wrote that sometimes she feels severe pain that makes her extremely nervous. A number of participants wrote that they can bear a finger pricking but they do not like needles for drawing their blood for INR testing.

Some participants showed their frustration with the possibility of taking OAT for life. A participant with PE was afraid and scared of a higher or lower level of his INR and wrote, "This has been a very scary experience no doubt!" A few of the participants with PE sometimes felt severe pain that made them extremely nervous; hence, they wanted to know more about the origin of the PE and the reasons behind the severe pain. One of them having PE and DVT wrote, "I've had some really weird and sharp pains. Not lasting very long though but it's quite scary."

Participants in blogs felt joy in sharing the experience and communicating with others bloggers. One of them wrote, "I am so glad to find this... and talk to others who have been through it. I have been solo for 2 years." In addition, they shared other social issues such as travelling abroad and the joy of visiting new places despite having health problems. They provided encouragement and advised each other to relax to avoid the stress, due to the illness, that might make the things worse. In addition, they appreciated each other such as a participant wrote, "Thanks for the advice and support."

## Discussion

The Internet in general and social media sites such as blogs in particular are increasingly becoming areas of academic interest because expressions of personal views and thoughts on key themes can be analysed by increasingly sophisticated methods. The Internet has provided a formal voice to those who may otherwise be unheard, or only heard locally. In addition, most of the people who have access to the Internet use it for either gaining information or exchanging their experiences on a wide range of issues such as healthcare and medical device technologies. Whilst there are always issues of representativeness in analysing such material the increasing ubiquity and availability of this medium to many, increases the opportunity for more widely applicable findings. It should also be noted in particular that those who may be disadvantaged by other means of social communication, through illness or disability, find considerable advantages in the use of such media.

The novelty of this study is that it focuses on the patients in relation to PT/INR self-testing whereas previous studies, discussed below, were focused on the device usability and reliability and the cost-effectiveness of self-testing compared to laboratory testing of PT/INR. This study has reported qualitative analysis of patients' experiences and opinions as revealed in their own narratives expressed, without any interference from the researcher, in the form of weblog postings regarding self-testing, also known as home testing, of OAT.

Generally, results show that the blogging patients shared their experience of PT/INR testing mainly in two biggest healthcare systems i.e. the US healthcare system and the NHS in the UK.

The findings reveal that the users, through their comments, see a number of advantages in the self-testing of OAT such as the saving of time, reducing travel, and reducing other costs involved in visiting health facilities for PT/INR testing; increasing control over their personal health and enhancing personal choice in the selection of the mode of the testing. This is in line with what has already been reported in the literature using a range of other methods. For example, patient self-monitoring provides independence and convenience to patients [2]. The advantages of PT/INR self-monitoring overall are often seen as including improving healthcare resource allocation by freeing time for both physicians and laboratory personnel and better management of OAT; thus, reducing chances of the complications directly associated with being outside the standard therapeutic range [3].

However, self-testing could not be undertaken by every patient on OAT but only by a tiny minority of patients [2], who qualify for the formal professional standards required to allow them to proceed to self-testing [41]. Even for those who qualify for self-testing there are requirements for training and quality control [3] as well as more generally encouragement, self-motivation to ensure adherence to protocols. Otherwise, patients might stop undertaking self-testing, which, amongst other consequences, would lead to a loss of the investment in the testing equipment and training. Whilst training may be provided in the form of a demonstration for several minutes, this might not be enough for some patients as indicated in the blogs' postings reported in this paper. Therefore, as indicated in other studies, patients need proper training both theoretical and practical to ensure the safety and effectiveness of the testing regime [5], which would almost certainly require standardized and monitored training procedures [2].

There are no exact figures of the numbers of patients undertaking self-monitoring of their INR. There are however consistent anecdotal reports that only 1-2% of people on OAT may be using coagulometers for INR self-testing, despite many attempts by manufacturers and a number of clinical centres to encourage their use. This relatively low acceptance and use of the devices by their intended patient users may be due to various reasons. As revealed from the patients' blogs reported in this paper, a major barrier to the uptake of PT/INR self-testing is the cost of both the device and testing strips [2].

Another factor limiting PT/INR self-testing could be the availability of a free home testing service undertaken by a community nurse in certain countries or areas within countries, as reported in the blogs analysed in this study. This however is more likely to occur where healthcare as a whole is provided free at the point of delivery such as through the NHS in the UK but this might not hold in other cases. The general lack of formal evidence of clinical safety and the effectiveness of patient self-testing compared to professional testing could be another limiting factor in the extension of self-testing [2]. This is likely to be compounded by the extensive and often well-developed infrastructure of hospital or clinical testing which, in some respects, self-testing may be seen as undermining, or indeed be being undertaken largely outside the monitoring or control of that infrastructure.

It was interesting to note that blogs' participants did not raise any issues relating to the design and usability of the device and test strips as well as errors or other problems encountered during PT/INR self-testing. It might be possible that the participants who used the equipment never encountered such problems or these issues were not raised on blog discussion boards. In this regard, a possible alternative explanation could be that these issues are already well addressed by the manufacturers before the equipment is deployed in the market.

Though reliable self-monitoring devices are available, there is an urgent need for agreed and widely available quality assurance mechanisms for self-monitoring [2]. In addition, there is a need to control pre analytical errors which occur in PT/INR monitoring at both home and the hospital [3]; otherwise patients might stop self-testing for economic, social and psychological reasons. Another issue that patients raised in their blogs was the availability of technical services for self-testing device calibration, which should be timely, regularly and standardised to ensure safety and confidence in the equipment both by patients and by those professionally monitoring device use [42].

According to Yang et al [3], manufacturers are responsible for device calibration because they control device calibration protocols [43]. However, patients' blogs show that they have been contacting their healthcare providers for the device calibration, where manufacturers have been less active or available in this respect. Therefore, patients show in the blogs that they need to know clearly whom to contact for device calibration, in particular through determining the roles of healthcare provider and device manufacturer, as determining who should bear the costs associated with the role.

Research studies have shown mixed findings in relation to the cost effectiveness of monitoring oral coagulations [44]. Lafata et al [45] have reported that patient self-monitoring (PSM) is not cost effective compared to health professional monitoring (HPM) while Taborski et al [46], Cheung et al [47] and Regier et al [48] reported that PSM is indeed more cost effective than the HPM. Overall, it is clear that further research needs to be undertaken on this issue to determine finally, what the position is. However, this study has demonstrated several reasons why there are likely to be continuing debates about the cost effectiveness of self-testing of oral coagulations. The issues of selecting and training patients as well as maintaining devices are critical [2,49]. Personal as well as social factors are similarly important, and what stands out from the data is that the notion of an entirely self-contained process of self-testing, where contact with manufacturers or healthcare system is minimal, is unlikely to materialise. From the patient blogs it is evident that very frequently there is the need for training (and retraining), monitoring, calibration, assessment or reassurance at all stages of self-testing particularly where a failure in any one of these areas could have damaging, if not fatal consequences for patients.

## Limitations of this study design

The authors recognise limitations of extracting information from blogs and using it for the academic research. The research design applied in this study used a set of key words to identify a suitable sample of blogs to collect the required data. In doing so, some relevant blogs might have been missed as well as some important issues regarding self-testing of OAT might have not been discovered through this study design. Therefore, in depth information about self-testing of OAT can be collected through other research designs such as the in-person interviews of an appropriate sample of patients. Moreover, another limitation of blogs is a lack of complete demographic details of bloggers, as reported in this study. Therefore, findings of this study may be interpreted cautiously and no attempt should be made to generalise these findings. However, the findings of this study can be recognised as an exploratory work, which can help in planning further studies as mentioned earlier.

## Conclusions

As indicated in this paper, INR testing currently involves a very large number of patients. Many of these patients are older, not least because the conditions, which result in the need for anticoagulation therapy as well as the associated INR testing, occur far more frequently with age. Formal healthcare resources needed for the continuing care of such patients are substantial. Thus, self-testing of INR levels should, in principle, substantially reduce these dedicated resources. Indeed, this argument is frequently the basis on which both manufacturers of self-testing devices as well as those concerned with the deployment of healthcare resources suggest the value of self-testing could be measured. However, this study, whilst primarily qualitative rather than quantitative in nature, suggests that the complexities of developing an effective self-testing programme are substantial.

Whilst it may be argued, rightly, that the analysis of Internet blogs is a problematic methodological endeavour for charting the exact dimensions of the issues in developing self-testing programmes, the data suggest the very wide range of issues that from a patient's point of view may determine their initial or continuing use of self-testing. Of course, some of these issues are very healthcare system specific, in this case, largely conditioned by the very different systems in the US and the UK. However, what is more surprising is the commonality of concerns, which seem to operate, almost independent of the healthcare system itself. As at a fundamental level for patients on anticoagulation therapy INR testing IS a matter of life or death (for too little or too much anticoagulation therapy may result in fatal consequences), many of the issues raised have this point at their core. Self-testing can be liberating from the drudgery of operating only with the pattern of (often poor or inconvenient) service provided by the healthcare system, as some bloggers have indicated, but, at the same time, there are frequently concerns about the effectiveness, reliability and use of self-testing equipment. Moreover, as appears to be indicated above, such concerns may not only be expressed by patients but also by healthcare professionals.

Whilst being very conscious of issues of the exact representativeness of those who choose to blog, as in the case of this paper, there is evidence from other studies to suggest that the range of issues covered in the blogs is sufficiently broad and all-embracing to give a consistent and clear picture of the extent of those issues, which are exercising 'self-testers'. In this respect, analysing such blogs may prove to be a useful first step in the preparation of more detailed face-to-face studies in this area, particularly where an analysis of such blogs proves both congruent and complementary to other studies using different methods.

## Competing interests

The authors declare that they have no competing interests.

## Authors' contributions

Both authors conceived the study, and participated in its design. SGSS collected and analysed the data and drafted the manuscript. IR guided the study, reviewed, and revised the manuscript. Both authors read and approved the final manuscript.

## Pre-publication history

The pre-publication history for this paper can be accessed here:

http://www.biomedcentral.com/1472-6963/11/25/prepub
